# Cholecystocolonic Fistulas from Diverticulosis: A Potentially Missable Cause of Liver Abscesses

**DOI:** 10.1155/2016/4803461

**Published:** 2016-11-22

**Authors:** Ben Warner, Terry Wong, Philip Berry

**Affiliations:** Gastroenterology, Guy's and St Thomas' NHS Foundation Trust, London, UK

## Abstract

Cholecystocolonic fistulas (CCF) due to colonic diverticulosis are a rare cause of liver abscesses. It is even rarer to simultaneously have choledocholithiasis, another cause for liver abscesses. In this case report, we found both pathologies and emphasise the need to study cholangiograms carefully so as not to miss alternative diagnoses.

## 1. Case Presentation

**Figure 1:**
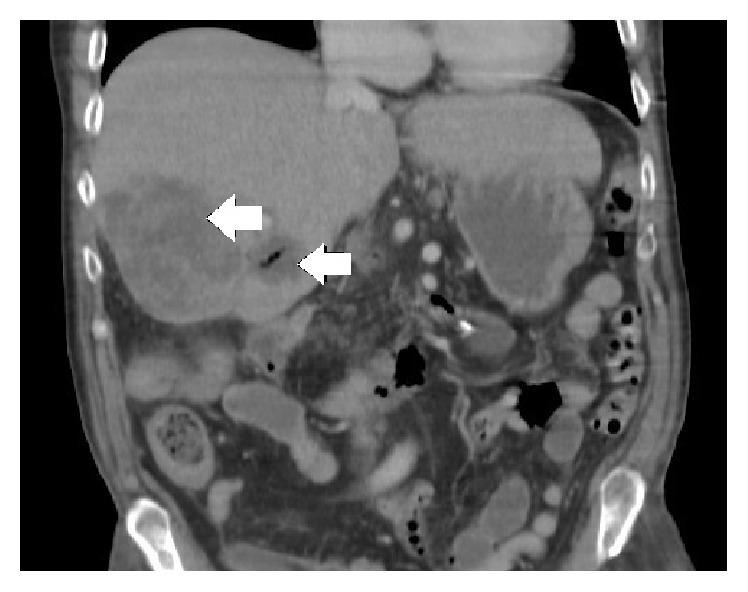
CT showing liver abscess formation within the liver.

An 82-year-old man with a history of chronic diarrhoea was admitted with severe Gram-negative sepsis, mild jaundice (bilirubin 60 *μ*mol/L), and coagulopathy (INR 1.9). Liver function tests were also deranged (alkaline phosphatase 190 IU/L, alanine transferase 84 IU/L). Computed tomography (CT) revealed an 8.8 cm by 8.2 cm liver abscess containing small amounts of gas, inflammatory stranding in both the regions of the gall bladder and hepatic flexure, and aerobilia (Figure 1). The abscess was drained percutaneously and aspirates cultured* Escherichia coli*.

**Figure 2:**
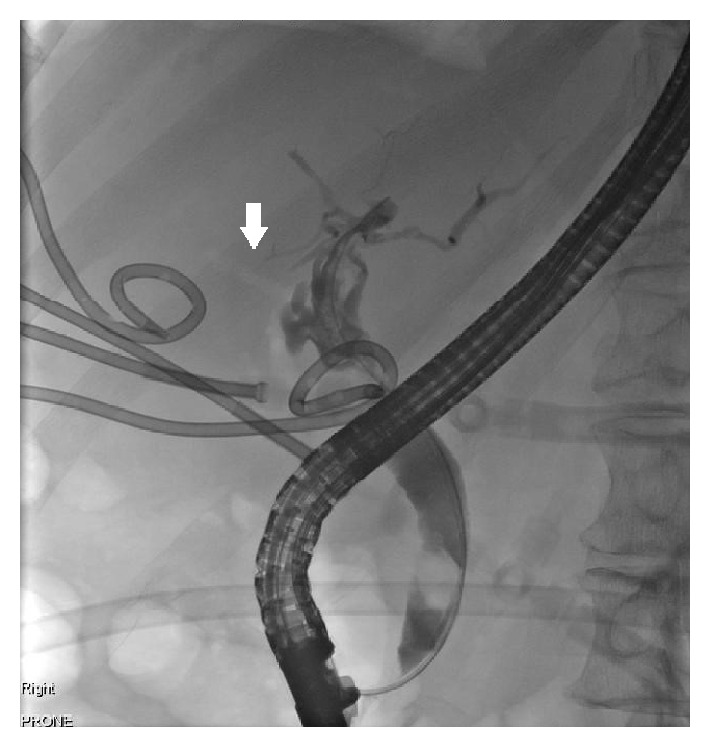
Cholangiogram at ERCP showing an 8 mm common bile duct stone with preferential filling of contrast of the left intrahepatic ducts and aerobilia (see arrow) on the right.

**Figure 3:**
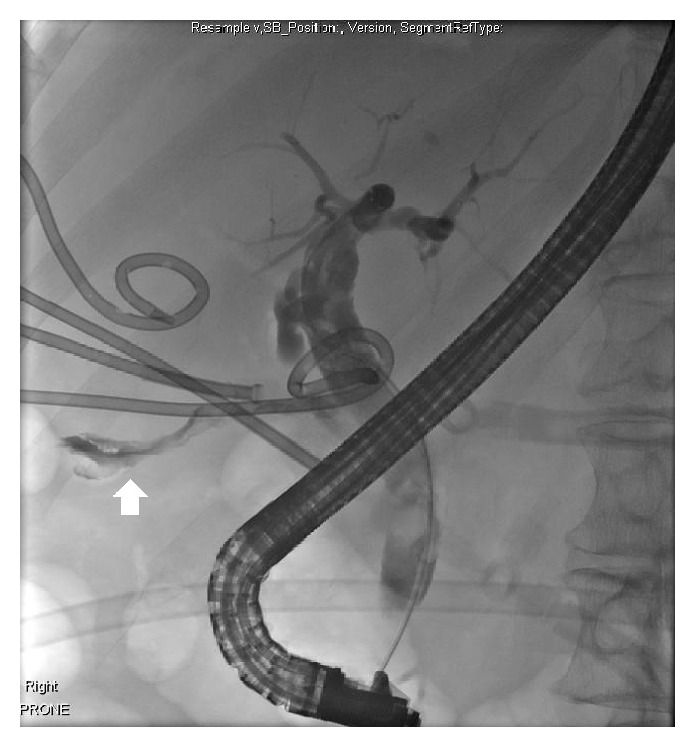
Cholangiogram showing collapsed gall bladder filling with contrast (see arrow).

**Figure 4:**
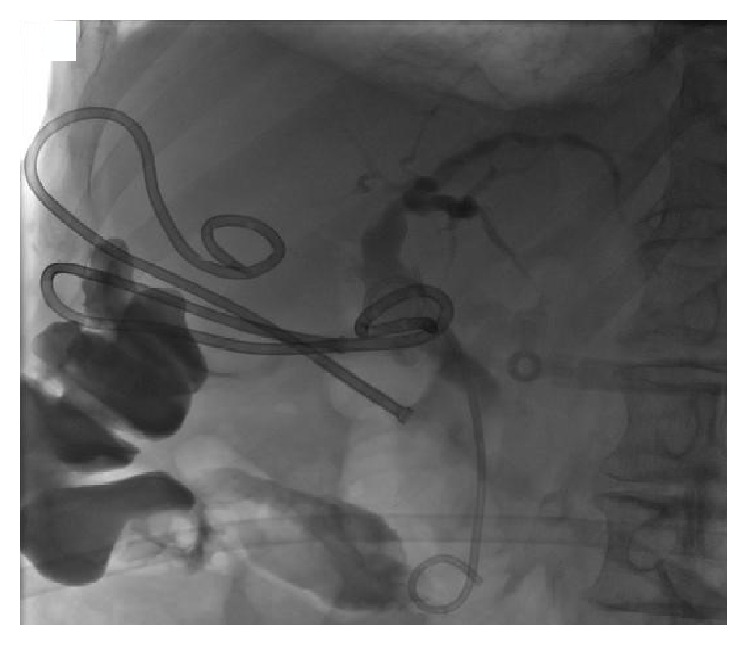
Cholangiogram showing contrast filling another viscus interpreted to be colon.

Magnetic resonance cholangiopancreatography (MRCP) showed a common bile duct stone. Endoscopic retrograde cholangiopancreatography (ERCP) demonstrated an 8 mm common bile duct stone with preferential filling of contrast of the left intrahepatic ducts and aerobilia (prior to sphincterotomy) on the right (Figure 2). Initially, the collapsed gall bladder filled with contrast (Figure 3), but in later images contrast collected in another viscus, interpreted to be colon (Figure 4). A sphincterotomy was performed, the stone was removed with a balloon trawl, and a double pigtail stent was inserted. Following identification of the CCF, the patient underwent a colonoscopy. This showed bile staining of the transverse and ascending segments. At the hepatic flexure there was significant diverticulosis, but no fistulous opening was seen, and tumour was excluded.

At surgery, the fistula was excisable and the opening to the colon was sealed with an omental plug. This, along with cholecystectomy, was performed as a one-stage procedure. Histology of the specimen confirmed the presence of an acutely inflamed fistula within the gall bladder with mucosal transition to large bowel mucosa. There was no dysplasia or malignancy detected. No gall bladder stones were found. The patient's recovery was complicated by a hospital acquired pneumonia from which he died 6 weeks later.

## 2. Discussion

The case is rare for the fact there were two potential causes of liver abscesses present. The presentation of sepsis, jaundice, and choledocholithiasis led us to presume that the abscess was caused by cholangitis and initial endoscopic management focussed on the stone's removal. Even after opacification of the colon at ERCP, we still felt the cause of the fistula to be biliary in origin.

CCFs occur in 0.06 to 0.14% of patients with biliary disease typically affecting females in their 6th and 7th decades [1]. Cholecystoduodenal fistulas, resulting in gall stone ileus or Bouveret Syndrome, are more common [1]. It is rare for CCFs to originate from the colon. For example, Crohn's disease does not fistulate to the gall bladder. Most CCFs instead develop due to gall bladder disease and gall stones, but colonic diverticulosis has been implicated [2]. In this case, the patient did not have gall bladder stones but he did have significant diverticulosis. Fistulation commonly occurs at the hepatic flexure due to its proximity to the gall bladder. Uncomplicated CCFs are mostly diagnosed intraoperatively presenting a challenge to surgeons who then need to convert to open cholecystectomy [1].

Diarrhoea is the most common presentation (71% of patients) due to the laxative effects of bile acids on the colon [1]. Our patient had experienced diarrhoea for some weeks prior to admission. In retrospect, the presentation of diarrhoea, coagulopathy (due to vitamin K malabsorption), and aerobilia, in the absence of a previous sphincterotomy, are a previously described triad pathognomonic of CCFs [3].

Gall bladder cancers can underlie some cases of CCF and frozen specimens should be examined for this postoperatively [1].

Endoscopic closure of these fistulas by over the scope clip system (OTSC) has been described [4]. The presence of diverticulosis made this method relatively contraindicated. ERCP and sphincterotomy are thought to encourage CCF closure although this is more relevant for cases caused by stone disease [5]. In our patient, it is possible that the combination of both a CCF and biliary stone accentuated the formation of an abscess due to inadequate biliary drainage as his jaundice resolved following the stone's removal.

Both surgical and conservative management approaches have been advocated, the latter in view of many patients' frailties or comorbidities. In uncomplicated CCF, a one-step procedure can be performed. For frail patients, a two-stage procedure involving a defunctioning colostomy is an option [1]. Either way, intervention is required if there is ongoing hepatobiliary sepsis to prevent further contamination from colonic contents. In our patient, it was felt that he required a definitive treatment. However, in retrospect he was frail following a long Intensive Care Unit admission and further physiotherapy and nutritional support could have been instituted, to improve his performance status prior to surgery.

## 3. Conclusion

The presence of the CCF may well have been a coincidental finding and not contributory to the abscess. On the other hand, the fistula, if left, could have led to further biliary sepsis. In conclusion, we highlight the importance of studying cholangiograms carefully for simultaneous pathologies. There are several potential causes for CCFs and a colonoscopy is advised to rule out the presence of diverticulosis or tumour which would require surgical intervention.
